# Ketamine modulates fronto-striatal circuitry in depressed and healthy individuals

**DOI:** 10.1038/s41380-020-00878-1

**Published:** 2020-09-14

**Authors:** Anahit Mkrtchian, Jennifer W. Evans, Christoph Kraus, Peixiong Yuan, Bashkim Kadriu, Allison C. Nugent, Jonathan P. Roiser, Carlos A. Zarate

**Affiliations:** 1 Section on the Neurobiology and Treatment of Mood Disorders, Experimental Therapeutics & Pathophysiology Branch, National Institute of Mental Health, National Institutes of Health, Bethesda, MD USA; 2 Neuroscience and Mental Health Group, Institute of Cognitive Neuroscience, University College London, London, UK; 3 Magnetoencephalography Core Facility, National Institute of Mental Health, National Institutes of Health, Bethesda, MD USA

**Keywords:** Depression, Diagnostic markers

## Abstract

Ketamine improves motivation-related symptoms in depression but simultaneously elicits similar symptoms in healthy individuals, suggesting that it might have different effects in health and disease. This study examined whether ketamine affects the brain’s fronto-striatal system, which is known to drive motivational behavior. The study also assessed whether inflammatory mechanisms—which are known to influence neural and behavioral motivational processes—might underlie some of these changes. These questions were explored in the context of a double-blind, placebo-controlled, crossover trial of ketamine in 33 individuals with treatment-resistant major depressive disorder (TRD) and 25 healthy volunteers (HVs). Resting-state functional magnetic resonance imaging (rsfMRI) was acquired 2 days post-ketamine (final sample: TRD *n* = 27, HV *n* = 19) and post-placebo (final sample: TRD *n* = 25, HV *n* = 18) infusions and was used to probe fronto-striatal circuitry with striatal seed-based functional connectivity. Ketamine increased fronto-striatal functional connectivity in TRD participants toward levels observed in HVs while shifting the connectivity profile in HVs toward a state similar to TRD participants under placebo. Preliminary findings suggest that these effects were largely observed in the absence of inflammatory (C-reactive protein) changes and were associated with both acute and sustained improvements in symptoms in the TRD group. Ketamine thus normalized fronto-striatal connectivity in TRD participants but disrupted it in HVs independently of inflammatory processes. These findings highlight the potential importance of reward circuitry in ketamine’s mechanism of action, which may be particularly relevant for understanding ketamine-induced shifts in motivational symptoms.

## Introduction

Over the past two decades, ketamine has emerged as a rapid-acting and potent antidepressant [1, 2]. However, the precise neural mechanisms underlying ketamine’s beneficial effects remain unknown. Unlike other antidepressants, ketamine is particularly effective in treating motivational dysfunction, such as anhedonia [3–5], a cardinal and treatment-resistant depressive (TRD) symptom [6]. In a parallel line of research, ketamine has also been used to model schizophrenia symptoms in healthy volunteers (HVs) [7]. Interestingly, some of those studies suggested that ketamine can transiently induce symptoms relating to impaired motivation in HVs [8–11]. This echoes our own findings that ketamine moderately increased anhedonia and symptoms of difficulty in decision-making in HVs beyond its dissociative side effects [12]. While this prior work suggests that ketamine’s effects may be mediated through changes in motivational processing, the neural circuit-level mechanisms underlying this are poorly understood.

A neural pathway of interest is the brain’s reward circuit, including striatum and ventral prefrontal cortex (PFC) [13]. The striatum acts as an important hub in the brain’s reward system and is thought to drive goal-directed behaviors through interplay with the PFC [14, 15]. For this reason, both theoretical and empirical accounts implicate the fronto-striatal circuit as a key driver of motivational behavior. In depressed individuals, task-based functional magnetic resonance imaging (fMRI) studies have consistently identified abnormalities in the brain’s reward system. Specifically, altered function has been observed in the ventral striatum (VS), orbitofrontal cortex (OFC), dorsolateral PFC (dlPFC), and perigenual anterior cingulate cortex (pgACC) [16–21]. Complementing and extending these findings, studies investigating resting-state fMRI (rsfMRI)—which is thought to reflect the intrinsic functional organization of neural circuits—reported that depression is associated with altered functional connectivity between striatal and prefrontal regions [22–28]. Furthermore, disrupted striatal and prefrontal function have been associated with individual differences in reward-related processing [29–33], suggesting that fronto-striatal circuitry plays an important role in the pathogenesis of motivational impairment.

Several inflammatory processes have recently been proposed to influence the function of this fronto-striatal circuit as well as motivational impairments in depression [34–36]. Elevated peripheral markers of inflammation—as measured by C-reactive protein (CRP)—have been associated with depression [37–39] and with lower cortico-striatal functional connectivity [29, 40]. Experimentally induced inflammation in animals and humans has also been shown to cause motivational impairments and reduce striatal function [41–44]. Inflammation may mediate motivational symptoms by dampening dopamine activity within reward circuitry, resulting in disrupted fronto-striatal functional connectivity [35]. Inflammatory processes are therefore well situated to influence neural circuits underlying motivational symptoms. Interestingly, ketamine may affect dopaminergic function through glutamatergic downstream effects [45, 46] and may also influence inflammatory processes [47, 48].

Although these studies lend credence to the hypothesis that fronto-striatal circuitry is important in ketamine’s mechanism of action, this has never been directly tested. A secondary question is whether ketamine-induced fronto-striatal changes are mediated via inflammatory mechanisms. These questions were explored in the context of this double-blind, placebo-controlled, crossover trial of ketamine in individuals with TRD and HVs that used rsfMRI to probe fronto-striatal circuitry and CRP measures to quantify peripheral inflammation. Given that ketamine has opposite effects on motivational symptoms in individuals with TRD and HVs, ketamine’s effects on reward circuitry and inflammation—two important neurobiological mechanisms underlying motivational behaviors—may underlie these observations. The hypothesis was that ketamine would increase functional connectivity within the fronto-striatal circuitry of TRD participants but decrease it in HVs, and that these effects would be associated with ketamine-induced changes in inflammatory response.

## Methods and materials

### Participants

Data for 58 participants (25 HVs and 33 TRD participants) were drawn from a larger clinical trial (NCT00088699). Inclusion and exclusion criteria were previously published [12, 49]. All TRD participants met criteria for recurrent major depressive disorder (MDD) without psychotic features, had a Montgomery–Åsberg Depression Rating Scale (MADRS; [50]) score ≥ 20 at screening and before each infusion, and had not responded to at least one adequate antidepressant trial during their current episode. Before testing, all TRD participants were medication-free for at least 2 weeks (5 weeks for fluoxetine, 3 weeks for aripiprazole). HVs had no Axis I disorder. Additional information can be found in the Supplementary (Tables S1 and S2). All participants provided written informed consent, and the study was approved by the NIH Combined Central Nervous System IRB.

### Study procedures

In this double-blind, placebo-controlled, crossover study, participants were randomized to receive either a single intravenous infusion of subanesthetic-dose ketamine hydrochloride (0.5 mg/kg) or placebo (0.9% saline solution) during the first session and the alternative treatment in the second session, conducted 2 weeks later. rsfMRI scans were obtained 2 days following each infusion. MADRS and Snaith–Hamilton Pleasure Scale (SHAPS, a measure of anhedonia [51]) ratings were acquired 60 min before each infusion and at 40, 80, 120, 230 min, and 1, 2, 3, 7, 10, and 11 days following each infusion.

### fMRI acquisition

Data acquisition and preprocessing were identical to those described in [49]; details are available in the Supplementary. Briefly, eight-minute rsfMRI scans (3.75 × 3.75 × 3.5 mm resolution, 64 × 64 matrix, repetition time (TR) of 2.5 s) were acquired on a 3 T GE Healthcare MRI scanner (HDX; Milwaukee, WI) with an eight-channel coil. Participants were asked to close their eyes and relax but not fall asleep.

### Seed regions

In line with previous studies [22, 29, 52], four striatal seeds reflecting striatal functional subregions were chosen to assess fronto-striatal circuitry (3.5 mm radius spheres). These included the VS (±9, 9, −8), dorsal caudate (DC; ±13, 15, 9), dorsal caudal putamen (DCP; ±28, 1, 3), and ventral rostral putamen (VRP; ±20, 12, −3). Left and right seeds were combined for analysis to increase signal-to-noise, as we hypothesized that left and right seeds would show similar activity. For each participant, seed locations were visually inspected with reference to anatomical images to ensure appropriate positioning.

### Region-of-interest (ROI) control

The primary visual cortex (V1) was used as a control region for a sensitivity analysis examining whether the results were specific to the identified PFC regions or due to a global pattern. Left and right ROIs (3.5 mm sphere radius per ROI) were collapsed for analysis (±8, −76, 10; [53]).

### Peripheral inflammatory biomarkers

CRP levels were used to assess peripheral inflammation. These were acquired 60 min prior to each infusion and at 230 min, Day 1, and Day 3 after each infusion. Only data from Day 1 were examined here as it was the timepoint both closest to the scan and infusion day and also had the greatest number of available samples. Acquisition and preprocessing details can be found in the Supplementary.

### Data analysis

Seed-to-whole-brain functional connectivity analyses were performed in AFNI (v.19.0.09) [54]. The final post-ketamine sample included 27 TRD participants and 19 HVs, and the final post-placebo sample included 25 TRD participants and 18 HVs (see Supplementary for additional details). Functional connectivity Fisher transformed z-maps were generated at the subject-level using 3dNetCorr [55]. Linear mixed-effects models were conducted (3dLME; [56]) at the group level to assess the effect of treatment on each seed region-to-whole-brain functional connectivity. Each model included: random effect of subject; within-subject factors of treatment (ketamine and placebo) and infusion order; and a between-subjects group factor (HV and TRD). Infusion order was retained if there were significant treatment interactions. Only results from the group-by-treatment interaction are presented here. An initial cluster-forming threshold of *p* < 0.005 (uncorrected) with cluster-level family–wise error (FWE) correction at *p* < 0.05 was used to correct for multiple comparisons. Monte-Carlo simulation in AFNI (3dFWHMx, 3dClustSim) yielded a minimum cluster size of 46 voxels. Significant clusters—derived from the group-by-treatment whole-brain analyses—were used in correlational analyses with symptoms and CRP measures as described below.

V1 control analyses, exploratory CRP analyses, and symptom analyses were conducted with SPSS (v25, IBM Corp, Armonk, NY). Linear mixed-effects analyses (random effect: subjects; fixed effects: group, treatment, and their interaction) were conducted to assess whether ketamine influenced striatal (VS, DC, DCP, VRP)-V1 functional connectivity. An identical linear mixed-effects model assessed the effect of ketamine on CRP levels. For this analysis, CRP measures were log-transformed to conform to assumptions of normality.

Pearson correlation coefficients explored the relationship between change in CRP measures and ketamine-induced shifts in fronto-striatal functional connectivity. Participants were included if they had CRP and rsfMRI data for both post-infusion days (ketamine and placebo). Changes in CRP levels (ketamine minus placebo; ΔCRP) were correlated with changes in functional connectivity (ketamine minus placebo; ΔFC) for each identified region from the seed-to-whole-brain functional connectivity result (i.e., the group-by-treatment interaction results). Correlations were conducted separately for each group.

Functional connectivity changes were further correlated with ketamine’s acute and longer-term anti-anhedonic or antidepressant effects in TRD. Differences in MADRS (ketamine minus placebo; ΔMADRS) and SHAPS (ketamine minus placebo; ΔSHAPS) scores on Day 2 (the rsfMRI scan day) and Day 10 were correlated with post-ketamine changes in fronto-striatal functional connectivity (ketamine minus placebo). For all analyses, statistical significance was assessed at *p* < 0.05, two–tailed. No *a priori* power analysis was performed because the present study was a secondary analysis of a clinical trial [12].

## Results

### Ketamine had opposite effects on fronto-striatal connectivity in TRD participants and HVs

**Fig. 1:**
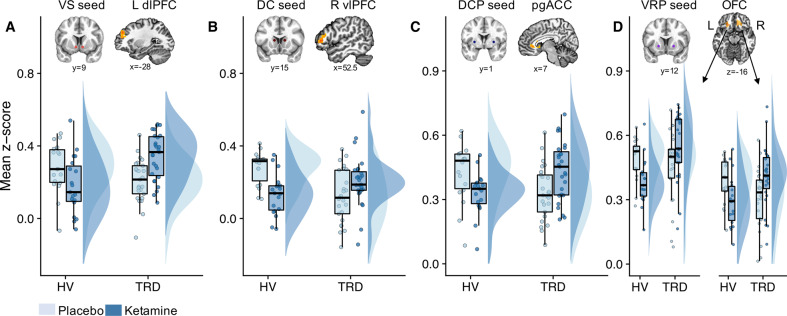
Group differences in the effects of ketamine on functional connectivity across four striatal seeds. Ketamine differentially altered functional connectivity between the groups, as reflected in VS-left dlPFC (**a**), DC-right vlPFC (**b**), DCP-pgACC (**c**), and VRP-left/right OFC (**d**) coupling. This was identified using group-by-treatment *F*–tests at a family-wise error (FWE) cluster-corrected threshold level of *p* < 0.05. Boxplots with individual data points and distributions [75] show that functional connectivity was increased in individuals with treatment-resistant depresssion (TRD) but reduced in healthy volunteers (HVs) post-ketamine relative to placebo (**a–d**). Resting-state functional magnetic resonance imagining scans (rsfMRI) were acquired 2 days after each infusion. VS ventral striatum; DC dorsal caudate; DCP dorsal caudal putamen; VRP ventral rostral putamen; dlPFC dorsolateral prefrontal cortex; vlPFC ventrolateral prefrontal cortex; pgACC perigenual anterior cingulate cortex; OFC orbitofrontal cortex; L left; R right; FWE family–wise error.

Significant group-by-treatment interactions were observed across all striatal seeds (Table 1). Specifically, functional connectivity between VS-left dlPFC, DC-right ventrolateral PFC (vlPFC), DCP-pgACC, and VRP-OFC was increased in TRD participants but decreased in HVs post-ketamine (Fig. 1). These results remained largely unchanged when controlling for potential confounds (Supplementary Table S3), although DCP connectivity may have been influenced by race. All other significant effects are presented in Supplementary Table S4. Ketamine’s group-specific effects are presented in Supplementary Table S5.

**Table 1:** Striatum-to-whole-brain functional connectivity results.

Effect	Seed	Label	Size (voxels)	Peak *x*	Peak *y*	Peak *z*	*F*-statistic	Alpha
Group × treatment	VS	Right putamen	79	21	5.2	−3.8	*F*_(1,36)_ = 27.06	<0.01
		Left dlPFC	51	−28	43.8	31.2	*F*_(1,36) _= 20.54	<0.04
	DC	Right vlPFC	52	52.5	36.8	3.2	*F*_(1,32) _= 20.37	<0.03
	DCP	pgACC	58	7	33.2	−0.2	*F*_(1,36) _= 17.18	<0.03
	VRP	Left OFC	81	−21	26.2	−10.8	*F*_(1,32)_ = 28.22	<0.01
		Right OFC	66	28	26.2	3.2	*F*_(1,32)_ = 16.96	<0.02

All clusters were corrected for multiple comparisons with a cluster-forming threshold of *p* < 0.005 (uncorrected) and family–wise error (FWE) cluster correction at *p* <  0.05 using Monte-Carlo simulation in AFNI.

*VS* ventral striatum, *DC* dorsal caudate, *DCP* dorsal caudal putamen, *VRP* ventral rostral putamen, *dlPFC* dorsolateral prefrontal cortex, *vlPFC* ventrolateral prefrontal cortex, *pgACC* perigenual anterior cingulate cortex, *OFC* orbitofrontal cortex.

### Ketamine’s group effects were specific to the fronto-striatal circuitry

**Fig. 2:**
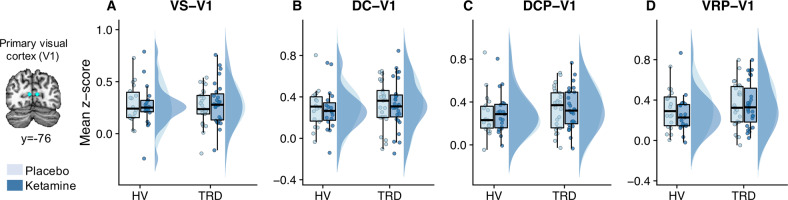
Ketamine had no effect on functional connectivity (data acquired 2 days post infusion) between the striatum and primary visual cortex (V1). Individual data points, boxplots, and data distributions are plotted for ventral striatum (VS)-V1 (**a**), dorsal caudate (DC)-V1 (**b**), dorsal caudal putamen (DCP)-V1 (**c**), and ventral rostral putamen (VRP)-V1 (**d**) functional connectivity post-ketamine and post-placebo for healthy volunteers (HVs) and individuals with treatment-resistant depression (TRD).

Control analyses indicated that ketamine exerted no observable effects on functional connectivity between any of the striatal seeds and the V1 control region (group-by-treatment interaction striatal-V1 functional connections: all *F*s < 1.99, all *p*s > 0.17; Fig. 2).

### Association with inflammatory biomarkers

No significant main effects on CRP levels were noted for group (*F*_(1, 48.50)_ = 1.11, *p* = 0.30), treatment (*F*_(1, 45.52)_ = 0.37, *p* = 0.55), or group-by-treatment interaction (*F*_(1, 45.52)_ = 1.61, *p* = 0.21; Supplementary Fig. S1).

**Fig. 3:**
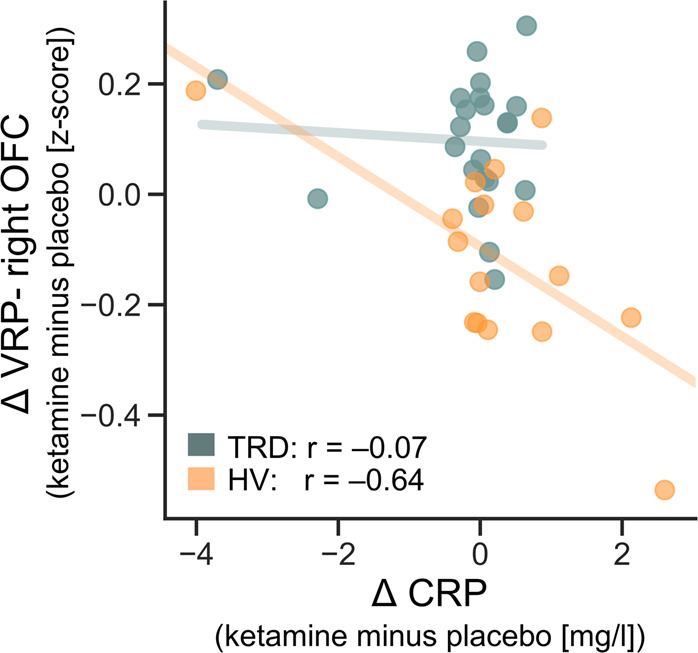
Association between post-ketamine changes in inflammation and functional connectivity. The relationship between changes in ketamine and peripheral inflammation (measured by C-reactive protein (CRP) 1 day post–infusion) with changes in functional connectivity (measured 2 days post–infusion) between the ventral rostral putamen (VRP) and right orbitofrontal cortex (OFC). Data are plotted separately for healthy volunteers (HVs; *p* = 0.007) and individuals with treatment-resistant depression (TRD; *p* = 0.77). Δ ketamine minus placebo.

A negative association was observed between ΔCRP and VRP-right OFC ΔFC in HVs (*r* = −0.64, *p* = 0.007; Fig. 3), such that increased CRP levels post-ketamine correlated with decreased VRP-right OFC functional connectivity. However, this was not the case for TRD participants (*r* = −0.07, *p* = 0.77; Fig. 3). The correlation coefficients between HVs and TRD participants did not differ significantly but were at trend level (Fisher’s *Z* test: *z* = 1.91, *p* = 0.06). No other relationships between ΔCRP and ΔFC post-ketamine were significant (all rs < −0.45, all *p*s > 0.08).

### Association with symptoms on Day 2

**Fig. 4:**
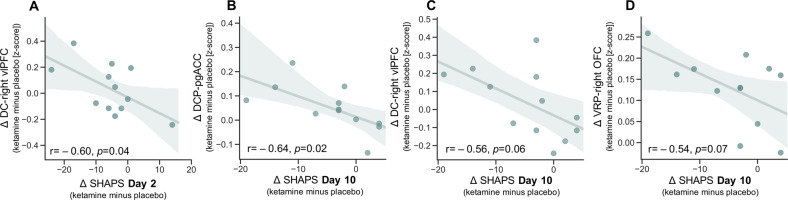
Association between post-ketamine changes in anhedonia and functional connectivity. Relationship between post-ketamine, compared with post-placebo, increases in fronto-striatal functional connectivity and improvements (negative numbers indicate post-ketamine improvements compared with post-placebo) in anhedonia symptoms on the day of the resting-state functional magnetic resonance imaging (rsfMRI) scan (2 days post infusion; **a**), and 10 days post infusion (**b**–**d**) in individuals with treatment-resistant depression (TRD). Shaded area represents estimated 95% confidence interval. SHAPS Snaith–Hamilton Pleasure Scale; DC dorsal caudate; vlPFC ventrolateral prefrontal cortex; DCP dorsal caudal putamen; pgACC perigenual anterior cingulate cortex; VRP ventral rostral putamen; OFC orbitofrontal cortex; Δ ketamine minus placebo.

No significant correlations were noted between ΔMADRS and ΔFC at Day 2 in TRD participants (all rs < −0.20, *p*s > 0.38). However, a significant correlation was observed between post-ketamine improvement (i.e., reduction) in SHAPS score and post-ketamine increases in DC-right vlPFC functional connectivity on Day 2 (Fig. 4a; *r* = −0.60, *p* = 0.04; all other ΔSHAPS and striatal-PFC ΔFC associations at Day 2: rs < 0.16, all *p*s > 0.62).

### Association with symptoms on Day 10

No significant correlations were observed between ΔMADRS and ΔFC at Day 10 in TRD participants (all rs < −0.32, *p*s > 0.18). Improvement in Day 10 SHAPS scores were associated with post-ketamine increases in DC-pgACC connectivity (Fig. 4b; *r* = −0.64, *p* = 0.02), and there was a trend toward a similar pattern for DC-right vlPFC connectivity (Fig. 4c; *r* = −0.56, *p* = 0.06) and VRP-right OFC (Fig. 4d; *r* = −0.54, *p* = 0.07). No other correlations between fronto-striatal ΔFC and Day 10 ΔSHAPS approached significance (Supplementary Fig. S2; all rs < −0.47, *p*s > 0.12).

## Discussion

This study, which sought to examine how ketamine affects fronto-striatal neural circuitry in TRD participants versus HVs, found that ketamine modulated fronto-striatal circuitry in a diagnosis-specific manner. In TRD participants, ketamine increased functional connectivity between the caudate and prefrontal regions (left dlPFC and right vlPFC) involved in cognitive processes and between the putamen and prefrontal regions (pgACC and OFC) associated with affective processes. However, in HVs, functional connectivity in these same frontal regions decreased post-ketamine. Notably, this was not simply due to a global shift in functional connectivity across the brain, as previously suggested [8], but was specific to the PFC [57]; in particular, striatal-visual cortex connectivity was not similarly affected by ketamine. These results underscore the complexities of ketamine’s neural effects.

Previous studies found that ketamine improves anhedonia symptoms and increases glucose metabolism in the VS, putamen, and dorsal ACC, extending into the sgACC and dlPFC, in individuals with treatment-resistant MDD and bipolar disorder [4, 5, 58]. Similarly, ketamine has been shown to increase striatal response during emotional processing [59] and global brain connectivity in the striatum and PFC [57]. The present study extends these findings by showing that, compared with placebo, ketamine improved functional connectivity within this fronto-striatal network in TRD participants. This is an important extension because psychiatric disorders may be better characterized as disruptions in circuit-level networks, given that many behaviors are achieved through multiple neural regions in concert [27].

While ketamine can improve motivational symptoms in depression, it often produces mild symptoms of impaired motivation, such as anhedonia and lassitude, in HVs [3–5, 8–12]. This pattern dovetails with our recent findings showing that ketamine restores dysfunctional neural mechanisms underlying emotional processing in depression but shifts these in the opposite direction in HVs [60, 61]. These diagnosis-dependent effects suggest that the initial functioning level of the neural circuit may be key to determining neurobiological response to ketamine. Interestingly, dysregulation of homeostatic neural mechanisms has been suggested to lead to altered functional connectivity in depression, particularly within cortico-limbic-striatal circuitry [20, 62]. Neuroplasticity models of ketamine’s beneficial effects suggest that ketamine may partly act by restoring disrupted homeostatic regulation [12, 62, 63]. The current results partially support this proposition, at least at the neural circuit level, given that increased anti-anhedonic effects were associated with greater functional connectivity post-ketamine between the DC and right vlPFC. If ketamine affects homeostatic neural regulation in general, this may also explain why ketamine may restore neural regulation in individuals with depression but disrupt fronto-striatal functioning in HVs. Ketamine also promotes glutamate signaling within cortico-limbic-striatal circuits and potentiates dopaminergic activity within the striatum and PFC [46, 62, 64], suggesting that glutamatergic signaling and downstream modulation of dopaminergic activity within the fronto-striatal circuitry may form a crucial part of ketamine’s neural effects [45, 59]. Future studies will need to directly examine whether the current findings stem from altered synaptic plasticity in reward circuitry.

Interestingly, increased fronto-striatal connectivity post-ketamine was associated with sustained improvements in anhedonia but not general depressive symptoms in TRD participants. The effects were most prominent for striatal interactions with the pgACC, although all PFC regions showed similar patterns (Fig. 4b–d and Supplementary Fig. S2). Changes in the brain’s reward system might therefore drive sustained motivational symptom improvements and could even serve as potential predictors of ketamine response, as suggested for other early signs of antidepressant response [65].

A secondary goal of the present study was to explore whether inflammatory processes, as assessed via CRP levels, affected ketamine-induced shifts in fronto-striatal connectivity [34–36]. Contrary to our hypotheses, no clear evidence suggested that ketamine-induced fronto-striatal connectivity changes depended on peripheral inflammatory processes. Increased CRP levels post-ketamine were associated with reduced VRP-right OFC functional connectivity, but only in HVs. This implies that downregulation of some aspects of the brain’s reward system may be associated with changes in inflammatory processes in HVs. This finding is in line with previous studies suggesting that inflammatory processes are particularly associated with OFC functioning [29, 40], although our OFC region was more lateral than found in previous studies. In addition, ketamine did not significantly affect CRP levels, nor did the association between change in VRP-right OFC and change in CRP levels post-ketamine differ significantly from the non-significant association in TRD participants. The identified association should therefore be considered tentative.

To date, ketamine’s effects on reward circuitry have not been extensively examined despite strong theoretical and empirical grounds [3–5, 62, 66, 67]. Echoing the present results, a previous study found reduced functional connectivity within cortico-striatal nodes in healthy nonhuman primates 24 h post-ketamine [68]. In contrast, another study found the opposite pattern in human HVs [69]. An important implication of the present study, however, is that investigations of ketamine’s antidepressant mechanisms should be interpreted with caution when based on healthy populations only, as previously reported by our group [12, 60, 61].

The present study adds to the growing literature suggesting disrupted fronto-striatal functioning in depression [16–24, 26, 27, 30–32, 70]. Both human and animal findings have suggested that fronto-striatal interactions are crucial for motivated behavior, i.e., integrating value signals with current goals to promote flexible goal-directed responding [13–15, 71]. Dysfunction in any of these processes could manifest as different depressive symptoms. Indeed, the prefrontal regions identified in the current study have been implicated in distinct aspects of goal-directed behavior. While the dlPFC and vlPFC have been shown to modulate cognitive control and flexible behavior, the OFC and pgACC have been implicated more directly in reward learning and decision-making [13, 14, 72]. For example, lower VS-dlPFC functional connectivity (identified here at placebo in TRD participants versus HVs) has been associated with impaired cognitive flexibility, while lower connectivity between the VS (including the VRP) and lateral OFC has been associated with poorer ability to flexibly update value representations to guide optimal decision-making [73, 74]. These findings suggest that ketamine’s fronto-striatal effects may be mostly related to flexible modulation over reward processes. Future studies should clarify the precise behavioral processes underlying ketamine-induced neural shifts.

A number of limitations merit comment. First, the rsfMRI scan occurred 2 days post infusion while ketamine’s beneficial effects occurred within hours of infusion; these effects have previously been shown to peak at 24 h post infusion [2, 4]. The sample size was also relatively modest due to challenges associated with a rigorous study design and patient population; these issues may have reduced our ability to properly characterize ketamine’s fronto-striatal effects. However, ketamine’s anti-anhedonic effects remained strong at the scanning timepoint in the current study, and this timepoint was chosen so that ketamine would be fully metabolized at the time of the rsfMRI scan, meaning that results would not be confounded by ketamine’s direct pharmacological effects. In addition, employing a within-subjects design provided greater statistical power because each individual acted as their own control. Nevertheless, future studies should aim to examine ketamine’s effects on fronto-striatal circuitry in larger sample sizes.

Second, the discrepancy between the rsfMRI timepoint and when the greatest beneficial effects of ketamine occurred (SHAPS Day 3; MADRS: 40 min; Supplementary Fig. S3) may also have reduced our sensitivity to properly characterize the relationship between post-ketamine fronto-striatal connectivity and symptom improvement. In particular, ketamine-induced increases in fronto-striatal connectivity would be expected to relate to improvements in anhedonia in TRD participants, but a significant relationship was detected only with DC-right vlPFC connectivity. That said, only a subset of the sample had SHAPS measures at both placebo and ketamine sessions, meaning that the study may have been underpowered to detect such associations. Similarly, the manner in which neural changes may relate to symptom changes in HVs was not investigated, given that all post-ketamine symptom changes had subsided to baseline levels by the day of the rsfMRI scan. However, the HV findings are consistent with previous studies demonstrating that decreased global connectivity in the striatum and decreased cerebral blood flow in the PFC are associated with increasing levels of negative symptoms/anhedonia immediately post-ketamine administration in HVs [8, 9]. Future studies should map ketamine’s neural effects across different timepoints and examine its relationship to different symptom dimensions. Nevertheless, these self-report measures were primarily constructed for clinical populations, were not designed to assess rapid symptom changes, and reflect a compound score of questions that may probe different cognitive and neural mechanisms. Thus, a more fruitful approach would be to examine associations with specific psychological processes using objective cognitive tasks.

Third, few participants had symptom and CRP data at both rsfMRI scans. Likewise, CRP and rsfMRI data were not available at the same timepoint. Due to the exploratory nature of these correlational analyses, the data were not corrected for multiple comparisons. As such, the symptom and CRP associations should be considered preliminary and require further confirmation.

Finally, in contrast to previous cross-sectional studies [37–39], the participant population did not differ from HVs in terms of baseline CRP levels (see Supplementary), suggesting that the current study captured a subgroup of TRD participants not characterized by dysfunctional inflammatory functioning. This may have obscured our ability to properly examine relationships with inflammation, as TRD participants did not exhibit a large range of CRP levels (Supplementary Figs. S1 and S4). Future studies should seek to recruit a more heterogeneous sample in terms of baseline inflammation levels to determine whether ketamine might exert important effects mediated by inflammatory processes.

In summary, the present study suggests that low fronto-striatal connectivity is normalized in TRD participants but disrupted in HVs post-ketamine, and that this occurs independently from peripheral inflammatory processes. This highlights the importance of including HVs as a normative model to draw comparisons. These findings support a homeostasis model of ketamine’s mechanism of action on functional network reconfiguration. Considering the crucial role that fronto-striatal circuitry plays in goal-directed behaviors, these findings may be particularly relevant for the rapid and sustained reorientation of motivational states observed post-ketamine.

## Supplementary information

Supplementary Materials

## Data Availability

All scripts used for analyzing data are available from the corresponding author upon request.
