# Supplementary material for: Ketamine modulates fronto-striatal circuitry in depressed and healthy individuals
Source: Mol Psychiatry. 2020 Sep 14;26(7):3292–301. doi: 10.1038/s41380-020-00878-1 (PMC8462973; doi:10.1038/s41380-020-00878-1)
Supplement: Supplementary file 1 — Supplementary Materials [file 41380_2020_878_MOESM1_ESM.docx]

# *Mkrtchian et al, “Ketamine modulates fronto-striatal circuitry in*

# *depressed and healthy individuals”*

**Supplementary Methods**

*Participant information*

All participants were evaluated using the Structured Clinical Interview for Axis 1 DSM-IV Disorders (SCID)-patient (1) and nonpatient versions (NP) (2). HVs had no family history of Axis I disorders in first-degree relatives as determined by the SCID-NP. All participants were deemed to be in good physical health with no unstable medical problems, as determined by medical history, physical examination, blood labs, chest x-ray, electrocardiogram, toxicology, and urinalysis. Information regarding participant characteristics can be found in Supplementary Table S1, and ketamine’s effects on Montgomery-Asberg Depression Rating Scale (MADRS) and Snaith-Hamilton Pleasure Scale (SHAPS, a measure of anhedonia (3)) scores can be found in Supplementary Table S2 and Supplementary Figure S3.

*Resting-state functional magnetic resonance imaging (rsfMRI) acquisition and preprocessing*

rsfMRI scans were obtained two days following each infusion; given ketamine’s short half-life (4), it had been fully metabolized by this time point, which allowed us to examine neural effects not attributable to ketamine’s immediate pharmacological effects.

Data acquisition and preprocessing were identical to (5). High-resolution structural images were obtained using a T1-weighted 3D fast spoiled gradient recalled echo (FSPGR) sequence with an 8.8s repetition time (TR), 3.4ms echo time (TE), 450ms inversion recovery time (IR), 13 degrees flip angle (FA), and with a 1mm isotropic resolution. Whole-brain rsfMRI images were obtained using a gradient recalled echo planar image (EPI) sequence with a 90˚ flip angle, 192 volumes, 45 slices per volume, 3.75x3.75x3.5mm resolution, 64x64 matrix, TR of 2.5s, TE of 25ms, anterior-posterior phase encoding direction and interleaved acquisition. In addition, cardiac and respiration traces were recorded during each scan using the GE photoplethysmograph and respiratory belt.

Preprocessing was accomplished in AFNI with the afni_proc script. This included despiking, slice-time correction, nuisance signal regression (motion: 12-parameter affine, registered to the third volume; physiological: slice-based, generated with McRetroTS), 6mm full-width-at-half-maximum (FHWM) spatial smoothing, band-pass filtering (0.01-0.1Hz), alignment to the Montreal Neurological Institute (MNI) 152 standard space, and motion censoring. Alignment to standard space was achieved by first aligning the structural image to the EPI with an affine transform using the LPC cost-function (align_epi_anat.py in AFNI). The anatomical image was non-linearly warped to the MNI 152 standard template and the EPI was transformed to standard space using the concatenated transformation matrices produced from the anatomical alignment steps. Image sequences were censored if there was movement greater than 0.2mm (Euclidean norm) per TR. If there were more than 15 censored time points per dataset, the dataset was excluded from further analysis. Motion (de-meaned and derivative) regressors were removed from the original time series simultaneously with band-pass filtering.

*Data information*

*fMRI data*

As reported previously (5), data were excluded for the following reasons: incomplete physiological data (six individuals with treatment-resistant depression (TRD) four healthy volunteers (HVs)); excessive motion (>0.2mm/TR; nine TRD, 11 HV); high correlation between the respiration volume trace and the average global signal, which increased correlations across the brain (three TRD, three HV); and an extreme outlier data point in the group x treatment interaction results (one HV). Functional connectivity analyses were thus conducted on 19 post-ketamine and 18 post-placebo HV scans and on 27 post-ketamine and 25 post-placebo TRD scans. Linear mixed-effects models were conducted on the rsfMRI scans obtained two days post-infusion. This analysis was chosen due to its advantages in analyzing mixed design data (between- and within-subjects) and because it allowed us to retain participants with only one post-infusion scan, thus increasing statistical power (6). Because no significant interaction was observed between infusion order and treatment for the VS and DCP seeds, order was removed from the final model for these two seed models.

*C-reactive protein (CRP) and symptom data*

Thirty-eight participants (TRD: 22, HV: 16) had usable C-reactive protein (CRP) and rsfMRI data at both post-infusion timepoints. Twenty-two TRD participants had MADRS scores at Day 2 after both infusions, and 12 TRD participants had SHAPS scores at Day 2 after both infusions. Nineteen TRD participants had MADRS scores at Day 10 after both infusions, and 12 had SHAPS scores at Day 10 after both infusions. The SHAPS is a 14-item, self-administered psychometric scale (3). Each item was scored between 1-4, resulting in a final score range between 14 and 56. For both scales, participants were asked to indicate how they felt since the last time the rating was administered. The primary symptom outcome was from Day 2, as this was the day of the rsfMRI scan. Secondary symptom outcomes were from Day 10, to explore if any ketamine-induced changes in fronto-striatal circuitry may have played a role in more sustained symptom improvements. For the correlations with the longer-term anti-anhedonic or antidepressant effects, psychometric data from Day 10 were chosen as this day had the greatest number of available samples.

*Inflammation biomarker acquisition and analysis*

The present study examined perhipheral and not central markers of inflammation, as these show high correspondence, suggesting that perhipheral CRP levels reflect central inflammation (7). Detailed analyses of ketamine’s effects on peripheral inflammatory biomarkers will be reported in a separate publication.

Blood samples were collected using BD vacutainer tubes with sodium heparin and centrifuged at 3000 rpm at 4°C for 10 minutes. Separated plasma samples were aliquoted and stored at -80°C until assay. Prior to processing, plasma samples were randomly allocated in the plates and blinded independently to minimize the impact of batch, treatment, or group effect in the sample. High-sensitivity CRP was quantified using the human CRP DuoSet ELISA kit (R & D Systems, Minneapolis, MN, USA) according to the manufacturer’s instructions. Plasma was diluted 1:1000 with reagent diluent and carried out in duplicate blind to clinical information. CRP standard solution was diluted to concentrations from 15.6 to 7500 pg/ml in order to create the standard curve. After the addition of biotinylated detection antibody, streptavidin-HRP substrate, and stop solution (stepwise), plates were read at 450 nm with Synergy HTX Multi-Mode Reader (BioTek, Winooski, VT, USA). CRP concentrations were calculated based on the standard curve.

CRP has previously been strongly positively associated with body mass index (BMI) (8-10). To examine if this was the case in the current sample, BMI was correlated with both raw and log-transformed CRP levels at placebo and ketamine for both groups. No significant correlations were observed between peripheral inflammation and BMI within the TRD (all r<0.37, all *p*>0.07) or HV groups (all r<0.41, all *p*>0.08).

An independent *t*-test was used to examine whether there were any baseline differences in CRP levels (log-transformed) between groups (TRD N=30, HV N=21). The -60 minute timepoint before the first infusion was used for the CRP data, as this most closely resembled approaches used in other cross-sectional studies investigating CRP levels between HVs and individuals with depression (8). No significant differences in CRP levels were found at baseline (t_(49)_=0.72, *p*=0.48; Supplementary Figure S4).

We also explored whether baseline CRP levels might moderate the change in fronto-striatal circuitry and anhedonia post-ketamine in individuals with TRD. The average of the log-transformed baseline CRP measures (-60 timepoint before both infusions) were correlated with change in SHAPS scores (ketamine minus placebo) as well as change in each identified striatal-frontal functional connectivity (ketamine minus placebo). A negative but non-significant relationship was observed between averaged baseline CRP levels and change in SHAPS scores post-ketamine (r=-0.14, *p*=0.61, n=15). No significant correlations were noted between averaged baseline CRP levels and ketamine-induced changes in fronto-striatal circuitry (all r=-0.17 to 0.35, all *p*>0.11, n=22).

*Clinical effects of ketamine*

A linear mixed effects model per group and symptom scale (MADRS, SHAPS) was used to examine the effect of ketamine versus placebo on symptoms (Supplementary Table S2, Supplementary Figure S3). Each model included a random effect for participants along with fixed effects of time, treatment, and their interaction. Baseline scores on each infusion day (-60 minutes) was included as a covariate to correct for baseline symptom levels.

*rsfMRI sensitivity analyses and results*

All striatal seed-to-whole-brain functional connectivity analyses were further examined when controlling for age, sex, race, and BMI. Linear mixed-effects models for each striatal seed were conducted in AFNI. Each model included: random effect of subject; group-by-treatment-by-order interaction and their main effects and lower interactions (order was only included for DC/VRP seeds where order significantly interacted with treatment in the original analysis); and main effects of each covariate and their interaction with treatment. Continuous covariates (age and BMI) were mean-centered within each group-by-treatment factor. Results are presented in Supplementary Table S3 and are largely unchanged from the main results. To explore what might be driving the discrepancy between DCP and VRP-right OFC results from the original results, separate DCP and VRP linear mixed models were conducted for each covariate. Results were largely unchanged from the main results (DCP and VRP seeds in Table 1) with separate sex, age, and BMI covariate models. However, in both the DCP and VRP models that included only the race covariate, the results mirrored the fully-adjusted effects (Supplementary Table S3), indicating that race was likely driving these deviations from the original results in Table 1. Because race was the only covariate that was unbalanced between groups (Supplementary Table S1), future studies should examine whether these effects remain when race is balanced between the groups. However, the original VRP-right OFC cluster (Table 1) overlapped with the covariate-controlled VRP-right OFC cluster (Supplementary Table S3), and although the VRP and DCP analyses including covariates differed slightly from the original results, it is important to note that each included covariate reduced the available degrees of freedom. Thus, the covariate-controlled results are less powered to assess ketamine’s effects on fronto-striatal circuitry.

**Supplementary References**

1. First MB, Spitzer RL, Gibbon M, Williams JB. Structured Clinical Interview for DSM-IV-TR Axis I Disorders, Research Version, Patient Edition (SCID-I/P). Biometrics Research, New York State Psychiatric Institute: New York, 2002.

2. First M, Spitzer R, Gibbon M, Williams J. Structured Clinical Interview for DSM-IV-TR Axis I Disorders, Research Version, Non-Patient Edition (SCID-I/NP). Biometrics Research, New York State Psychiatric Institute: New York, 2002.

3. Snaith RP, Hamilton M, Morley S, Humayan A, Hargreaves D, Trigwell P. A scale for the assessment of hedonic tone the Snaith-Hamilton Pleasure Scale. *Br J Psychiatry* 1995; **167**: 99-103.

4. Clements JA, Nimmo WS, Grant IS. Bioavailability, pharmacokinetics, and analgesic activity of ketamine in humans. *J Pharm Sci* 1982; **71**: 539-542.

5. Evans JW, Szczepanik J, Brutsche N, Park LT, Nugent AC, Zarate CA, Jr. Default mode connectivity in major depressive disorder measured up to 10 days after ketamine administration. *Biol Psychiatry* 2018; **84**: 582-590.

6. Chen G, Saad ZS, Britton JC, Pine DS, Cox RW. Linear mixed-effects modeling approach to FMRI group analysis. *Neuroimage* 2013; **73**: 176-190.

7. Felger JC, Haroon E, Patel TA, Goldsmith DR, Wommack EC, Woolwine BJ *et al.* What does plasma CRP tell us about peripheral and central inflammation in depression? *Mol Psychiatry* 2018; **Jun 12 [epub ahead of print]**.

8. Chamberlain SR, Cavanagh J, de Boer P, Mondelli V, Jones DNC, Drevets WC *et al.* Treatment-resistant depression and peripheral C-reactive protein. *Br J Psychiatry* 2019; **214**: 11-19.

9. Ridker PM, Buring JE, Cook NR, Rifai N. C-reactive protein, the metabolic syndrome, and risk of incident cardiovascular events: an 8-year follow-up of 14 719 initially healthy American women. *Circulation* 2003; **107**: 391-397.

10. Zhao Y, Lv G. Correlation of C-reactive protein level and obesity in Chinese adults and children: a meta-analysis. *J Endocrinol Invest* 2013; **36**: 642-647.

11. Nugent AC, Ballard ED, Gould TD, Park LT, Moaddel R, Brutsche NE *et al.* Ketamine has distinct electrophysiological and behavioral effects in depressed and healthy subjects. *Mol Psychiatry* 2019; **24**: 1040-1052.

| **Supplementary Table S1.** Characteristics for participants with at least one post-infusion (ketamine or placebo) scan included in the rsfMRI analyses. | | | |
| --- | --- | --- | --- |
|  | **TRD (n=30)** | **HV (n=21)** | ***p*-value** |
|  | **Mean (std. dev.)** | **Mean (std. dev.)** |  |
| Age | 36 (9.54) | 34 (10.97) | 0.55 |
| Female | 18 (60%) | 14 (67%) | 0.63 |
| BMI (kg/m^2^) | 26.54 (5.66) | 27.87 (4.16) | 0.37 |
| Race (Caucasian) | 25 (83%) | 11 (52%) | 0.02 |
| Length of illness | 20.80 (10.74) years | – |  |
| Length of current episode | 45.50 (73.20) months | – |  |
| Number of failed antidepressant treatments | 6.5 (3.66) | – |  |
| BMI: body mass index; rsfMRI: resting-state functional magnetic resonance imaging; TRD: treatment-resistant depression; HV: healthy volunteer | | | |

| **Supplementary Table S2.** Ketamine’s symptom effects | | | |
| --- | --- | --- | --- |
|  | **Main effect of treatment** | **Main effect of minutes** | **Treatment x minutes interaction** |
| **TRD: MADRS** | F_(1, 237.00)_=142.87, p<0.001 | F_(9,151.77)_=1.36, p=0.21 | F_(9, 92.98)_=1.08, p=0.38 |
| **TRD: SHAPS** | F_(1, 118.23)_=40.93, p<0.001 | F_(9, 82.71)_=9.86, p=0.57 | F_(9, 40.34)_=0.83, p=0.60 |
| **HV: MADRS** | F_(1, 290.10)_=33.45, p<0.001 | F_(9,285.11)_=11.23, p<0.001 | F_(9,282.97)_=7.68, p<0.001 |
| **HV: SHAPS** | F_(1, 208.77)_=2.52, p=0.11 | F_(9, 207.46)_=0.85, p=0.56 | F_(9, 207.17)_=1.10, p=0.37 |
| TRD: treatment-resistant depression; HV: healthy volunteer; MADRS: Montgomery-Åsberg Depression Rating Scale; SHAPS: Snaith-Hamilton Pleasure Scale. | | | |

| **Supplementary Table S3.** Striatum-to-whole-brain functional connectivity results controlling for sex, age, race and BMI | | | | | | | | |
| --- | --- | --- | --- | --- | --- | --- | --- | --- |
| ***Effect*** | ***Seed*** | ***Label*** | ***Size (voxels)*** | ***Peak x*** | ***Peak y*** | ***Peak z*** | ***F-statistic*** | ***alpha*** |
| Group * treatment | VS | Right putamen | 63 | 21 | 5.2 | -3.8 | *F*_(1,30)_=27.19 | <0.02 |
|  |  | Left dlPFC | 54 | -28 | 47.2 | 17.2 | *F*_(1,30)_=23.40 | <0.03 |
|  | DC | Right vlPFC | 43 | 52.5 | 36.8 | 3.2 | *F*_(1,28)_=22.51 | <0.07* |
|  | VRP | Left OFC | 85 | -21 | 26.2 | -10.8 | *F*_(1,28)_=28.57 | <0.01 |
|  |  | Right striatum/ OFC | 133 | 14 | 19.2 | -10.8 | *F*_(1,28)_=28.09 | <0.01 |
| Abbreviations: VS: ventral striatum; DC: dorsal caudate; VRP: ventral rostral putamen; dlPFC: dorsolateral prefrontal cortex; vlPFC: ventrolateral prefrontal cortex; OFC: orbitofrontal cortex. All clusters were corrected for multiple comparisons with a cluster-forming threshold of *p*<0.005 (uncorrected) and family-wise error (FWE) cluster correction at *p<*0.05 using Monte-Carlo simulation in AFNI resulting in minimum 46 voxels.  *This effect narrowly misses the minimum cluster-corrected voxel size of 46 voxels when all covariates are included but emerges at 47 voxels (*p*<0.05 FWE cluster-corrected) with sex excluded from the model. Sex did not exert a significant main effect or treatment interaction in the DC seed model, but its inclusion decreases degrees of freedom. | | | | | | | | |

| **Supplementary Table S4.** Significant results from each striatal whole-brain functional connectivity analysis | | | | | | | | |
| --- | --- | --- | --- | --- | --- | --- | --- | --- |
| **Effect** | **Seed** | **Label** | **Voxels** | **Peak x** | **Peak y** | **Peak z** | **F-statistic** | **alpha** |
| Treatment  (Ket > Pla) | VS | Precuneus | 71 | -0.0 | -71.8 | +48.8 | F_(1,49)_=17.00 | <0.01 |
| Group* Infusion order | DC | Insular cortex | 62 | +42.0 | -1.8 | -21.2 | F_(1,32)_=21.71 | <0.02 |
|  | DC | Frontal pole | 123 | +7.0 | +61.2 | -21.2 | F_(1,32)_=19.30 | <0.01 |
| Treatment* Infusion order | DC | Precuneus | 120 | +3.5 | -54.2 | +17.2 | F_(1,32)_=20.88 | <0.01 |
|  | VRP | Frontal pole | 154 | -0.0 | +61.2 | -3.8 | F_(1,32)_=26.88 | <0.001 |
| All clusters were corrected for multiple comparisons with a cluster-forming threshold of *p*<0.005 (uncorrected), with a family-wise error (FWE) correction at *p*<0.05 using Monte-Carlo simulation in AFNI. VS: ventral striatum; DC: dorsal caudate; VRP: ventral rostral putamen; Ket: ketamine; Pla: placebo. | | | | | | | | |

| **Supplementary Table S5.** Significant post-hoc group-specific ketamine effects from each striatal whole-brain functional connectivity analysis | | | | | | | | |
| --- | --- | --- | --- | --- | --- | --- | --- | --- |
| ***Treatment-resistant depressed (TRD) individuals*** | | | | | | | | |
| **Effect** | ***Seed*** | ***Label*** | ***Size (voxels)*** | ***Peak x*** | ***Peak y*** | ***Peak z*** | ***z-score*** | ***alpha*** |
| *Ketamine>*  *Placebo* | VS | Precuneus | 168 | -7 | -78.8 | 52.2 | 3.62 | <0.001 |
|  |  | Left OP10/dlPFC | 118 | -21 | 57.8 | 6.8 | 3.78 | <0.01 |
|  |  | Right OP10/ dlPFC | 104 | 24.5 | 43.8 | 24.2 | 4.64 | <0.01 |
|  |  | PCC | 96 | -3.5 | -26.2 | 31.2 | 4.27 | <0.01 |
|  | DC | Left OP10/dlPFC | 67 | -35 | 40.2 | 10.2 | 3.47 | <0.02 |
|  | VRP | dlPFC | 63 | -31.5 | 33.2 | 31.2 | 3.43 | <0.02 |
|  |  | Right OFC | 47 | 10.5 | 29.8 | -14.2 | 3.80 | <0.05 |
| ***Healthy volunteers (HV)*** | | | | | | | | |
| **Effect** | ***Seed*** | ***Label*** | ***Size (voxels)*** | ***Peak x*** | ***Peak y*** | ***Peak z*** | ***z-score*** | ***alpha*** |
| *Placebo>*  *Ketamine* | DC | Right vlPFC | 52 | 56 | 33.2 | 13.8 | -4.76 | <0.04 |
|  | VRP | Right SFG | 70 | 14 | -8.8 | 62.8 | -3.94 | <0.01 |
| Abbreviations: VS: ventral striatum; DC: dorsal caudate; VRP: ventral rostral putamen; dlPFC: dorsolateral prefrontal cortex; PCC: posterior cingulate cortex; vlPFC: ventrolateral prefrontal cortex; OFC: orbitofrontal cortex; SFG: superior frontal gyrus. All clusters were corrected for multiple comparisons with a cluster-forming threshold of *p*<0.005 (uncorrected) and family-wise error (FWE) cluster correction at *p<*0.05 using Monte-Carlo simulation in AFNI. There were no significant clusters from the dorsal caudal putamen seed in TRD or HV individuals, and no significant clusters from the VS seed in HVs at this threshold. Only ketamine>placebo contrast clusters were present for TRD patients and placebo>ketamine contrast clusters in HVs. | | | | | | | | |

**Supplementary Figure S1.** The effect of ketamine on peripheral inflammation, measured by C-reactive protein (CRP) levels (one day post-infusion) in healthy volunteers (HVs) and individuals with treatment-resistant depression (TRD). Note that raw CRP levels are presented but analyses were conducted on log-transformed data.

**
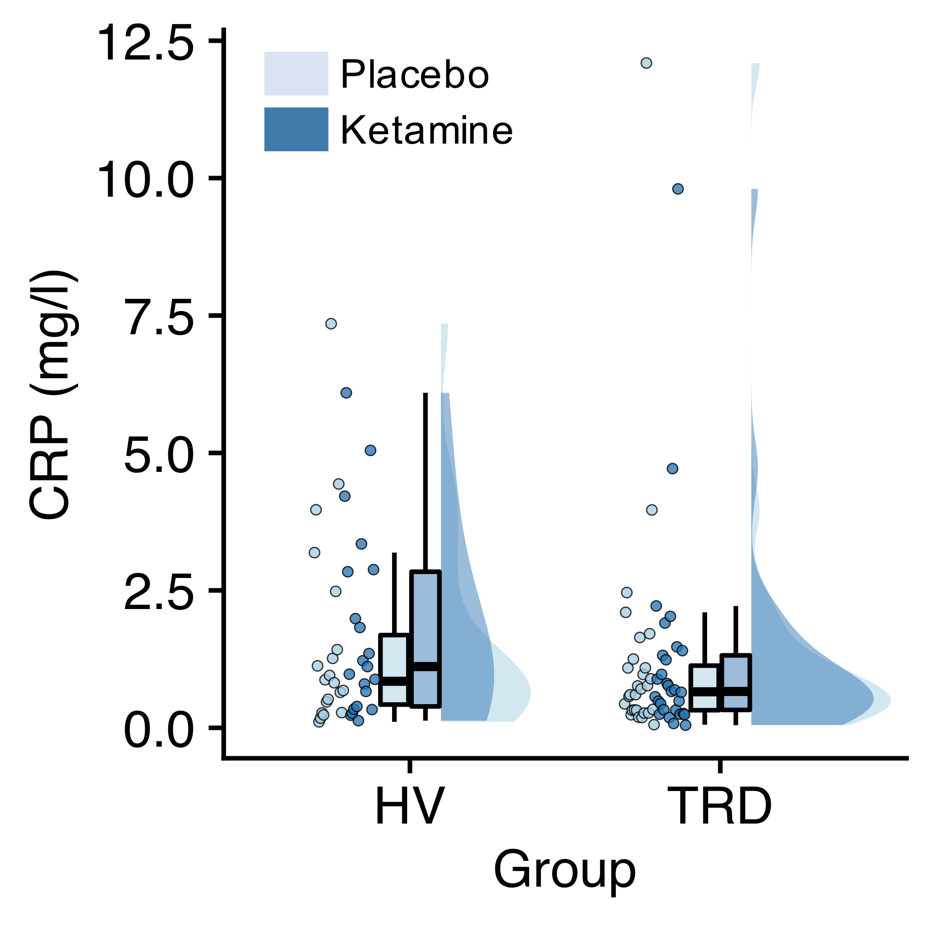
**

**Supplementary Figure S2.** Plots A and B show the relationship between post-ketamine change in ventral striatum (VS)-left dorsolateral prefrontal cortex (dlPFC) and ventral rostral putamen (VRP)-left orbitofrontal cortex (OFC) functional connectivity (measured two days post-infusion) with improvements in Snaith–Hamilton Pleasure Scale (SHAPS) score on Day 10 post-infusion in individuals with treatment-resistant depression (TRD). Shaded area represents estimated 95% confidence interval. Δ: ketamine minus placebo.

**
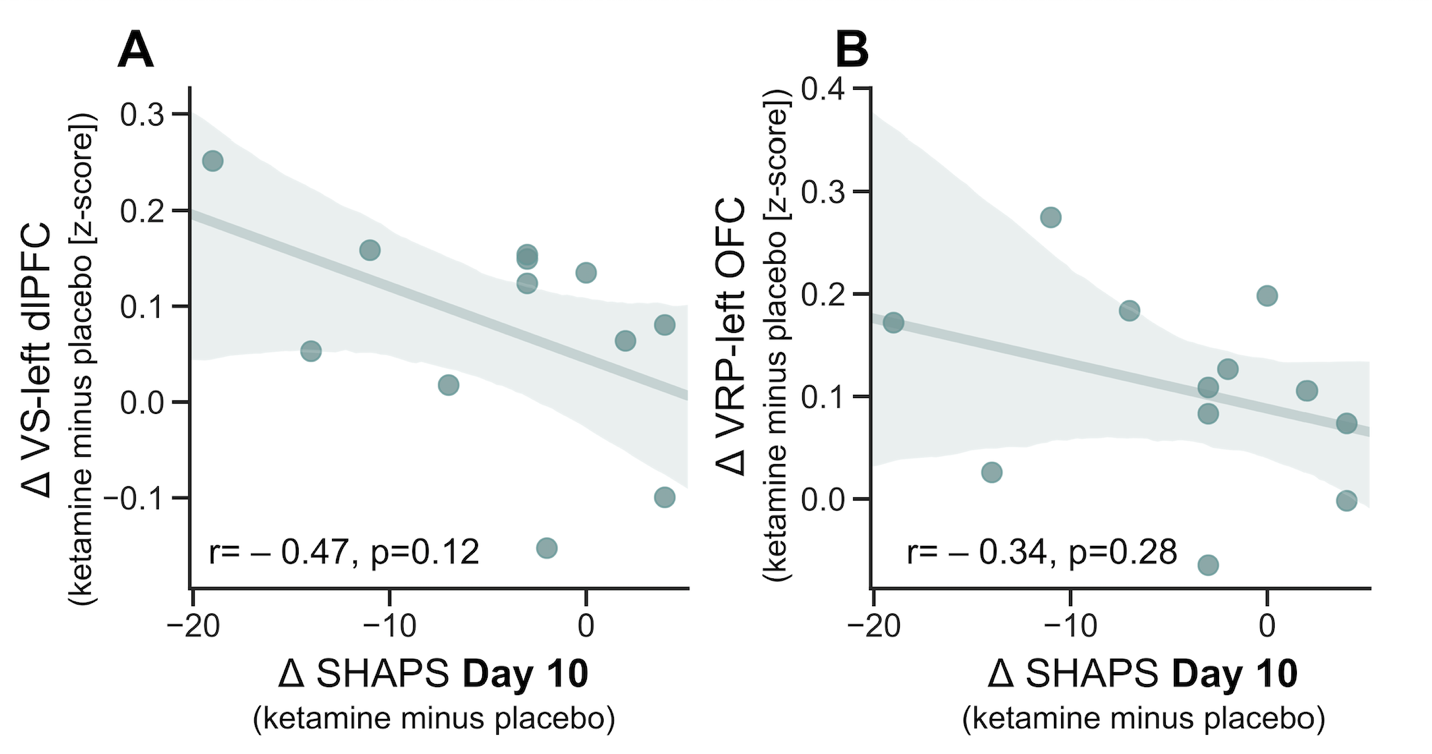
**

**Supplementary Figure S3.** Ketamine’s effects on symptoms within the current study sample. Participants represent a subsample drawn from a larger study (11). Plots A and B plot ketamine’s effects on Montgomery-Asberg Depression Rating Scale (MADRS) and Snaith-Hamilton Pleasure Scale (SHAPS) scores in individuals with treatment-resistant depression (TRD), and plots C and D plot these for healthy volunteers (HVs).

**
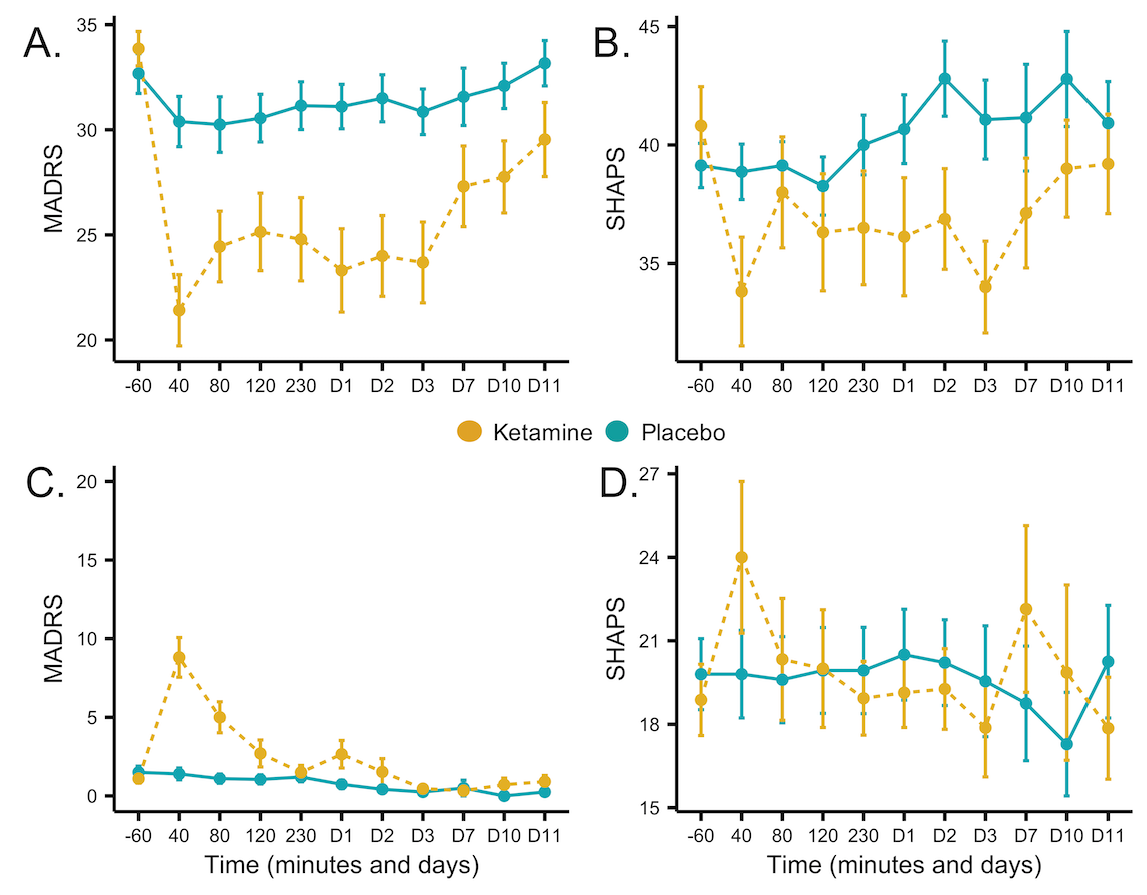
**

**Supplementary Figure S4.** C-reactive protein (CRP) levels in healthy volunteers (HVs) and individuals with treatment-resistant depression (TRD) at baseline (-60 min timepoint before first infusion). Note that raw CRP levels are presented but analyses were conducted on log-transformed data.

**
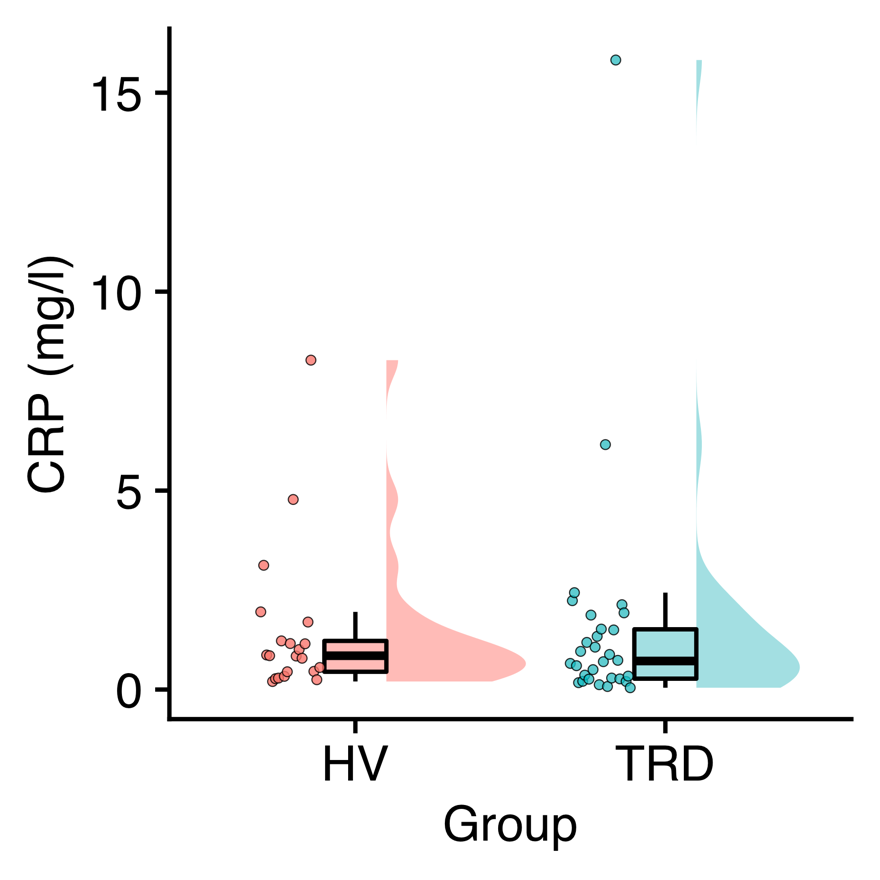
**
